# Intraoperative transesophageal echocardiography under aortic arch branch blood flow monitoring in a patient with an aberrant left subclavian artery: a case report

**DOI:** 10.1186/s40981-025-00828-2

**Published:** 2025-11-03

**Authors:** Yasutaka Suzuki, Takahiro Kawaji, Hidefumi Komura, Satoshi Komatsu, Naohide Kuriyama, Tomoyuki Nakamura

**Affiliations:** Department of Anesthesiology and Critical Care Medicine, Fujita Health University School of Medicine, 1-98 Dengakugakubo, Kutsukake-Cho, Toyoake, Aichi 470-1192 Japan

**Keywords:** Liver transplantation, Aberrant subclavian artery, Transesophageal echocardiography, RSO₂ monitoring, Arterial pressure monitoring, Compression risk

## Abstract

**Background:**

Transesophageal echocardiography (TEE) is widely used in cardiac and non-cardiac surgeries with major hemodynamic fluctuations. In patients with vascular anomalies near the esophagus, such as an aberrant left subclavian artery (ALSA), TEE may pose a risk of vascular compression. However, no guidelines exist.

Case presentation.

A 19-year-old woman with biliary atresia underwent living donor liver transplantation. Preoperative imaging showed a right-sided aortic arch with ALSA coursing posterior to the esophagus, and ALSA compression was considered a risk during TEE. Therefore, bilateral radial arterial pressures and regional cerebral oxygen saturation (rSO₂) were monitored. The TEE probe was inserted under videolaryngoscopic guidance without resistance or hemodynamic disturbance, and no arterial waveform attenuation or rSO₂ changes occurred. Transplantation was uneventful, and the patient showed no postoperative neurological or upper-limb deficits.

**Conclusion:**

This case highlights that appropriate imaging and monitoring strategies can support safe TEE use in non-cardiac surgery, even with vascular anomalies.

## Background

Transesophageal echocardiography (TEE) is a useful tool in cardiac anesthesia, including for risk management of complications and surgical decision-making [1]. TEE is now widely used not only in cardiac surgery but also in non-cardiac surgeries where drastic hemodynamic changes are expected or in cases of unexplained intraoperative hypotension [2]. Although TEE is considered minimally invasive, caution is required as probe insertion and manipulation may cause oropharyngeal, esophageal, or gastric trauma [3]. In particular, in patients with vascular anomalies adjacent to the esophagus, such as an aberrant subclavian artery (ASA), there may be an increased risk of vascular compression or injury [4–11]. However, no guidelines exist regarding preventive strategies. Here, we report a case of a living donor liver transplantation in a patient with an aberrant left subclavian artery (ALSA), in whom perioperative TEE during anesthesia was safely performed under arch branch blood flow monitoring.

## Case presentation

**Fig. 1:**
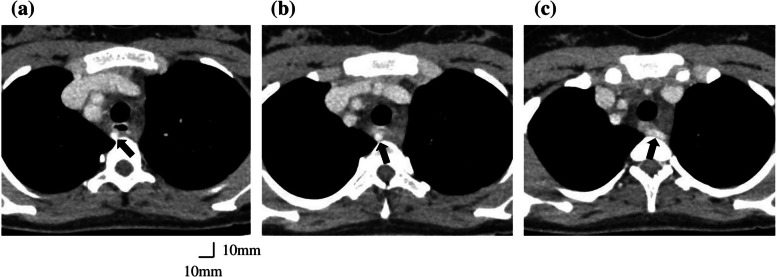
Thoracic contrast-enhanced computed tomography images. (→): Aberrant left subclavian artery

**Fig. 2:**
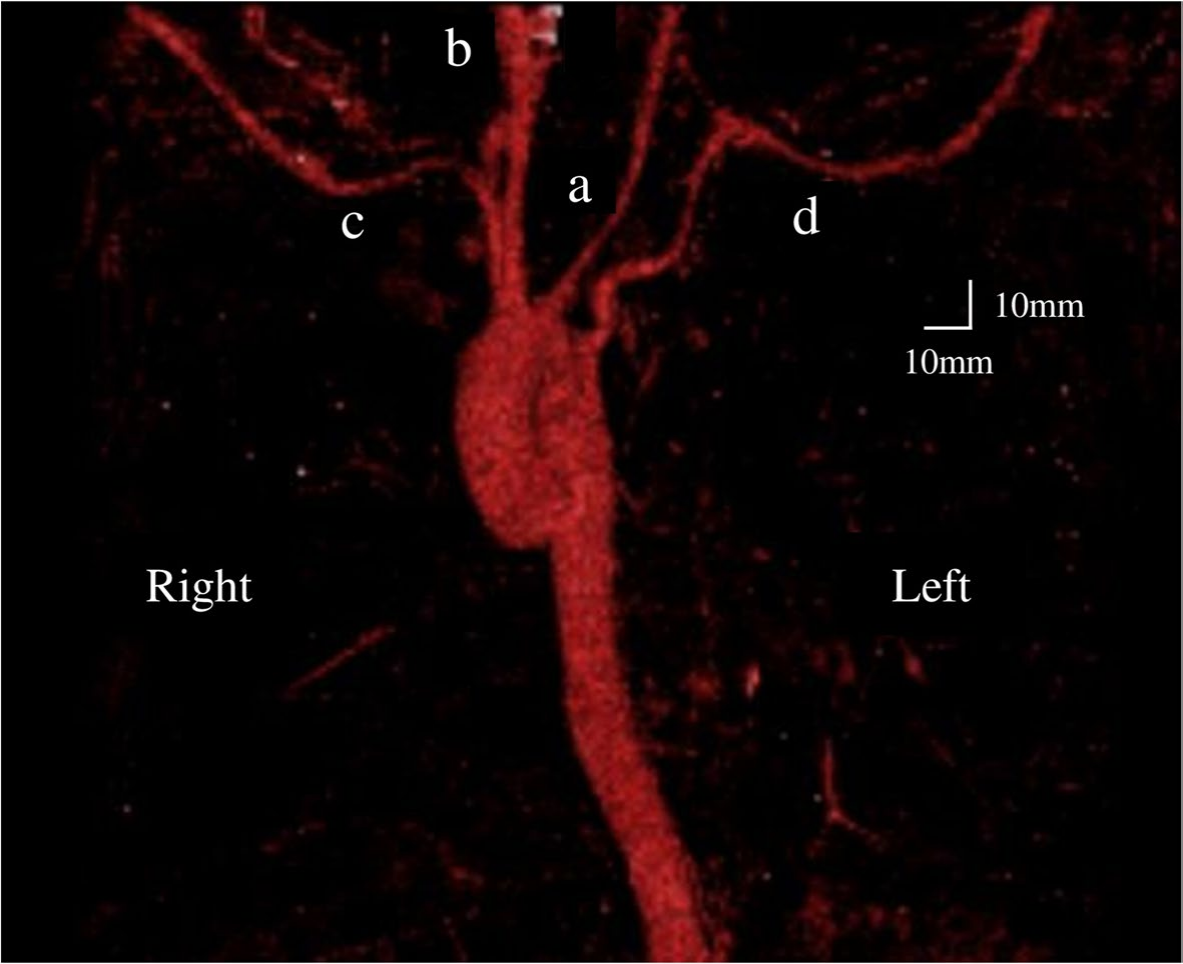
Three-dimensional computed tomography with vascular reconstruction. The left common carotid (**a**), right common carotid (**b**), right subclavian (**c**), and left subclavian arteries (**d**) branched sequentially from the proximal segment of the right-sided aortic arch. The left common carotid artery is narrower than the right common carotid artery. The left subclavian artery arises from the most distal portion of the right sided aortic arch and courses posterior to the esophagus, consistent with an aberrant left subclavian artery

A 19-year-old woman (height, 152 cm; weight, 50 kg) with congenital biliary atresia underwent Kasai portoenterostomy shortly after birth. Postoperatively, jaundice was well controlled, and she was regularly followed up. However, in recent years, she developed recurrent cholangitis and progressive cirrhosis, including massive splenomegaly, making it necessary to undergo a living-donor liver transplantation with her older sister as the donor. Preoperative contrast-enhanced CT demonstrated a right-sided aortic arch with a left subclavian artery coursing between the esophagus and vertebral column (Fig. 1). Three-dimensional vascular reconstruction revealed sequential branching of the left common carotid, right common carotid, right subclavian, and left subclavian arteries from the proximal segment of the right-sided aortic arch (Fig. 2). The left common carotid artery was narrower than the right common carotid artery. The left subclavian artery originated from the most distal segment of the right-sided aortic arch and coursed posterior to the esophagus, identifying it as ALSA. The patient had no symptoms of dysphagia. Although the patient had previously undergone endoscopic variceal ligation for esophageal varices, preoperative upper gastrointestinal endoscopy revealed no varices with red color signs and no obvious protruding lesions.

Given the risk of pronounced bleeding—particularly owing to adhesions from prior Kasai portoenterostomy and massive splenomegaly—we planned intraoperative TEE for careful hemodynamic monitoring. Pulmonary artery catheterization and advanced arterial pressure waveform analysis were considered as alternatives, but TEE was selected as it provides direct, real-time anatomic and functional cardiac assessment. However, because the ALSA coursed posterior to the esophagus, we anticipated a risk of vascular compression during TEE probe insertion and manipulation. Therefore, we added bilateral radial arterial pressures and regional cerebral oxygen saturation (rSO2) monitoring to detect compression-related changes. Worrisome attenuation of the left radial waveform or cerebral desaturation during probe insertion would have prompted immediate probe withdrawal and a switch to pulmonary-artery–catheter–based monitoring.

The patient underwent intraoperative monitoring, including electrocardiography, peripheral oxygen saturation, non-invasive blood pressure, processed electroencephalography (EEG) monitoring with SedLine® (Masimo Corp., Irvine, CA, USA), and rSO₂, which was measured using the O3® Regional Oximetry system (Masimo Corp., Irvine, CA, USA). Baseline rSO₂ values were 52% on the right and 59% on the left. General anesthesia was induced with 60 mg of propofol, remifentanil at 0.1 μg/kg/min, fentanyl 100 μg, and rocuronium 50 mg, using a rapid sequence induction and intubation technique. Following tracheal intubation, invasive arterial catheters were placed in both radial arteries, revealing a 15–20 mmHg lower pressure in the left radial artery compared to the right. A triple-lumen central venous catheter for drug administration and a 12-Fr sheath introducer for large-volume fluid infusion were inserted via the right internal jugular vein. The TEE probe (X7-2t transducer; Philips Healthcare, Andover, MA, USA; tip dimensions approximately 17 mm × 13 mm) was inserted under videolaryngoscopic guidance with careful visualization of the esophageal inlet. No resistance was encountered during insertion, and no significant changes were observed in left radial artery pressure or left-sided rSO₂. Blood flow assessment of ALSA was attempted using TEE, but assessment distal to the stenotic site was limited. General anesthesia was maintained with 3.5% desflurane, remifentanil at 0.4 μg/kg/min, and rocuronium at 7 μg/kg/min, along with noradrenaline at 0.05–0.1 μg/kg/min as a vasopressor. Throughout the intraoperative period, TEE manipulation did not cause attenuation or loss of the left radial arterial pulse waveform, nor did it result in significant rSO₂ differences between the right and left sides. In addition, the patient state index, a processed EEG parameter, remained stable at approximately 30. The procedure was prolonged due to adhesion dissection and hepatic artery reanastomosis. However, there were no major complications, including those related to the anhepatic phase or hepatic ischemia–reperfusion injury, and the surgery was successfully completed as planned. The TEE probe was removed postoperatively without blood contamination. The total durations of surgery and anesthesia were 1,362 and 1,217 min, respectively. Estimated blood loss was 5,414 g, and urine output was 2,900 mL. The patient received 4,100 mL of crystalloid, 2,250 mL of 5% albumin, 2,800 mL of packed red blood cells, 6,720 mL of fresh frozen plasma, 150 mL of cryoprecipitate, and 400 mL of platelet concentrate.

Postoperatively, the patient was transferred to the intensive care unit (ICU) under sedation with propofol at 4 mg/kg/h. Upon emergence from anesthesia, she exhibited no neurological deficits or upper limb dysfunction. Her postoperative course was uneventful; she was extubated on postoperative day 4 and discharged from the ICU on postoperative day 8.

## Discussion

This case shows that TEE can be performed safely in patients with an aberrant subclavian artery (ASA), despite a potentially increased risk of vascular compression. Preoperative imaging and intraoperative monitoring provided an extra safety net, confirming the absence of compression and ensuring that immediate corrective action would have been possible.

ASA is a congenital vascular anomaly in which the subclavian artery arises abnormally from the aortic arch and passes adjacent to the esophagus, predisposing it to compression by a TEE probe. Eight previous reports (five pediatric, three adult) described ASA compression during TEE, all showing loss of ipsilateral radial arterial waveform that resolved after probe withdrawal [4–11] (Table 1). The narrower esophagus in infants presumably increased their risk of compression. Of the five pediatric cases, four involved ARSA—including two with Down syndrome (trisomy 21)—and one involved ALSA with a right-sided aortic arch. Among the three adult cases, two involved ARSA and one ALSA associated with a right-sided aortic arch. ARSA is frequently associated with Down syndrome, particularly in patients with congenital heart disease [12], while ALSA is typically linked to a right-sided aortic arch and is rarer [13]. ASA was identified preoperatively in four of the five pediatric cases but in only one of the three adult cases (Table 1), suggesting underrecognition in adult non-cardiac surgery.

**Table 1:** Review of the literature on aberrant subclavian artery (ASA) compression during transesophageal echocardiography (TEE)

Author/year Reference No	Age	Comorbidities	Surgical procedure	Types of ASA	Monitoring	Diagnosis of ASA compression	TEE probe maneuver
Bensky AS/1995 [4]	5 months	Down syndrome	AVSD repair	ARSA	Right radial arterial pressure	Waveform dampened,, recovered after TEE withdrawal Confirmed by normal ascending aortic pressure	Continued
Koinig H/2003 [5]	7 weeks	Down syndrome	AVSD repair	ARSA	Right radial arterial pressure Contralateral NIBP and pulse oximetry	Waveform loss, recovered after TEE withdrawal Contralateral normal	Removal
Park SY/2010 [6]	75 years	Coronary artery disease	Off-pump coronary artery bypass graft	ARSA	Right radial arterial pressure Right femoral arterial pressure	Waveform loss, recovered after TEE withdrawal Femoral normal	Changed to smaller probe
Sierra RL/2011 [7]	62 years	Hepatocellular carcinoma	Hepatectomy	ARSA	Right radial arterial pressure and pulse oximetry Contralateral NIBP and pulse oximetry	Waveform loss, recovered after TEE withdrawal Contralateral normal	Removal
Garg V/2012 [8]	8 months	VSD tricuspid regurgitation	VSD repair	ARSA	Right radial arterial pressure Contralateral (leg) NIBP and pulse oximetry	Waveform loss, recovered after TEE withdrawal Contralateral (leg) normal	Removal
Lee WI/2013 [9]	1 years	TOF	TOF toal corrective surgery	ALSA	Left radial arterial pressure Femoral arterial pressure and contralateral pulse oximetry	Waveform loss, recovered after TEE withdrawal Femoral and contralateral normal	Removal
Hattori K/2016 [10]	6 months	AVSD	VSD closure	ARSA	Right radial arterial pressure Contralateral pulse oximetry	Waveform loss, recovered after TEE withdrawal Contralateral normal	Changed to smaller probe
Apatov DA/2020 [11]	54 years	Aortic stenosis History of CoA	Aortic Valve Replacement	ARSA	Right and left radial arterial pressure Contralateral NIBP	Waveform loss, recovered after TEE withdrawal Contralateral normal	Continued

*AVSD* Atrioventricular Septal Defect, *ARSA* Aberrant Right Subclavian Artery, *NIBP* Non-Invasive Blood Pressure, *VSD* Ventricular Septal Defect, *TOF* Tetralogy of Fallot, *ALSA* Aberrant Left Subclavian Artery, *CoA* Coarctation of the Aorta

In our case, preoperative contrast-enhanced CT revealed ALSA and narrowing of the left carotid artery. Because the circle of Willis provides only limited collateral flow [14], ischemia of the left arm and left cerebral hemisphere was a concern. Bilateral radial arterial pressures and regional cerebral oxygen saturation (rSO₂) were continuously monitored during TEE probe insertion. Although rSO₂ is less sensitive for vertebrobasilar ischemia [15], it is clinically useful for detecting cerebral hypoperfusion [16]. As an alternative, transcranial Doppler could be considered for evaluating vertebral artery blood flow; however, its continuous intraoperative use was deemed impractical. Therefore, rSO₂ was selected in the present case. To our knowledge, rSO₂ monitoring has not previously been reported in TEE-related ASA compression, and its potential value, demonstrated by the present study, warrants further investigation.

The 2010 ESC guidelines recommend preoperative imaging in patients with aortic or esophageal disease, but offer no specific intraoperative monitoring strategies [1]. Furthermore, growing evidence supports the safety and utility of TEE in liver transplantation anesthesia [17–20]. In these various settings, targeted monitoring of arch branch perfusion—as performed in the current study—may help detect otherwise unrecognized ASA compression and guide timely probe adjustment or removal.

As TEE use expands in non-cardiac surgery, the potential for unrecognized ASA compression increases, underscoring the importance of preoperative vascular assessment. While ASA may pose a risk during TEE, our case suggests that with appropriate imaging and monitoring, TEE can be used safely even in patients with arch anomalies.

## Data Availability

Not applicable.

## Abbreviations

ASA: Aberrant subclavian artery
ALSA: Aberrant left subclavian artery
ARSA: Aberrant right subclavian artery
CT: Computed tomography
rSO_2_: Regional saturation of oxygen
SpO_2_: Peripheral oxygen saturation
TEE: Transesophageal echocardiography
